# Exploring Market State and Stock Interactions on the Minute Timescale

**DOI:** 10.1371/journal.pone.0149648

**Published:** 2016-02-22

**Authors:** Lei Tan, Jun-Jie Chen, Bo Zheng, Fang-Yan Ouyang

**Affiliations:** 1 Department of Physics, Zhejiang University, Hangzhou 310027, China; 2 Collaborative Innovation Center of Advanced Microstructures, Nanjing 210093, China; University of Warwick, UNITED KINGDOM

## Abstract

A stock market is a non-stationary complex system. The stock interactions are important for understanding the state of the market. However, our knowledge on the stock interactions on the minute timescale is limited. Here we apply the random matrix theory and methods in complex networks to study the stock interactions and sector interactions. Further, we construct a new kind of cross-correlation matrix to investigate the correlation between the stock interactions at different minutes within one trading day. Based on 50 million minute-to-minute price data in the Shanghai stock market, we discover that the market states in the morning and afternoon are significantly different. The differences mainly exist in three aspects, i.e. the co-movement of stock prices, interactions of sectors and correlation between the stock interactions at different minutes. In the afternoon, the component stocks of sectors are more robust and the structure of sectors is firmer. Therefore, the market state in the afternoon is more stable. Furthermore, we reveal that the information of the sector interactions can indicate the financial crisis in the market, and the indicator based on the empirical data in the afternoon is more effective.

## Introduction

In recent years, the complex systems have drawn much attention of scientists in various fields, since the systems widely exist in the world of science. Financial markets are important examples of complex systems. Large amounts of historical financial data allow physicists to quantitatively investigate the properties in the markets with concepts and methods in statistical mechanics. Plenty of results have been obtained [1–20].

The stock interactions in complex financial systems are usually explored by investigating the equal-time cross-correlation matrix *C* of price returns [11, 12, 21–26]. With the random matrix theory (RMT), communities can be identified from *C*, and they are usually associated with industry sectors [23–25]. A number of large eigenvalues of *C* significantly deviate from the upper bound of the eigenvalue distribution of the Wishart matrix, which is the cross-correlation matrix of non-correlated time series. For examples, the number of such eigenvalues is 5 for the Shanghai stock market. For the largest eigenvalue, the components of the eigenvector are basically uniform and of a same sign for all stocks, which represents the collective dynamic behavior of the prices of all stocks. Thus, this eigenvalue stands for the market mode, i.e., the price co-movement at the market level. For the eigenvector corresponding to every other large eigenvalues, the absolute values of the components are significantly larger for stocks in a certain industry sector [11, 19, 25, 26]. Therefore, the eigenvector is dominated by this sector, and represents the collective dynamic behavior of stocks in the sector. Each of these eigenvalues corresponds to a sector mode, which is the price co-movement at the sector level. The market mode and sector mode may respectively arise from that stocks sharing common information of the market and that of the sector [19]. The RMT may identify the industry sectors in a financial market, but can hardly describe the interactions between sectors. Recently, the sector interactions are investigated with various methods in complex networks, such as the planar maximally filtered graph (PMFG), minimal spanning tree (MST) and map of information flow (Infomap) [27–31].

A stock market is a non-stationary complex system, and the market state evolves with time. To study the price dynamics in the system, we endeavor to investigate the market state. To the best of our knowledge, the price co-movement, e.g. the market and sector modes, and the interaction structure of sectors may reflect the market state. On the daily timescale, various activities have been devoted to the price co-movement and sector structure [11, 19, 25–33]. However, these two properties on the minute timescale may not remain the same. Since a market or sector is formed by stocks through the stock interactions, the price co-movement and sector interactions essentially originate from the interactions of stocks. The stock interactions are important for the understanding of the market state. For the properties of a financial market on the minute timescale, there have been many studies on the “intraday pattern” [3, 34–39]. The intraday pattern mainly concerns the dynamic behavior of a single index or stock near the opening and closing time of the market. Thus, our knowledge on the stock interactions on the minute timescale is still limited.

Financial crises have been of great interest to scientists and investors [40–46]. It is reported that the first eigenvalue of the cross-correlation matrix *C*, representing the market mode, can be an indicator for the financial crises, and the more significantly this eigenvalue changes, the more likely a financial crisis would occur [46]. However, it is hard to tell how much change in the first eigenvalue would indicate a financial crisis accurately. To obtain a reliable indicating, one needs more indicators.

In this paper, we investigate the dynamics of the stock interactions, and explore the market state on the minute timescale. Further, we construct an indicator based on the information of the sector interactions to indicate the financial crisis in the market.

## Materials

From “Wind Financial Database” (http://www.wind.com.cn), we collect the minute-to-minute price data of 200 stocks in the Shanghai stock market, most of which are large-cap stocks. The data of every stock are from July 27, 1999 to November 5, 2003. The Shanghai stock market is open from 9:30 to 11:30 in the morning, and from 13:00 to 15:00 in the afternoon. For each stock, since the price data are recorded every minute, there are 242 data points in a day, with 121 data points in both the morning and afternoon. There are 1014 trading days and 245388 data points for one stock. The total number of the price data of all stocks is about 50 million. Also, we collect the daily closing prices of the Shanghai Index from “Yahoo! Finance” (http://finance.yahoo.com) with the same period as that of the minute data, i.e. from July 27, 1999 to November 5, 2003.

## Results

On every trading day *t*, the trading minute is denoted by *τ*. For example, *τ* = 9: 30 is the opening time of the market, and *τ* = 15: 00 is the closing time. For each stock, there are 242 data points of prices in one trading day. The price of the *i*-th stock on day *t* at minute *τ* is denoted by *P*_*i*_(*t*, *τ*), and the logarithmic price return is *R*_*i*_(*t*, *τ*) = ln[*P*_*i*_(*t*, *τ*)/*P*_*i*_(*t*′, *τ*′)]. The time interval between (*t*, *τ*) and (*t*′, *τ*′) is denoted by Δ*t*, and Δ*t* = 242 ⋅ (*t* − *t*′) + (*τ* − *τ*′) minutes. Due to the Epps effect [47–49], the equal-time correlation of two stocks increases with Δ*t* and gradually converges when Δ*t* is larger than one hour. Here for the equilibration of the correlation between any two stocks, we set Δ*t* = 1 day, i.e., *R*_*i*_(*t*, *τ*) = ln[*P*_*i*_(*t*, *τ*)/*P*_*i*_(*t* − 1, *τ*)].

According to the trading minute *τ*, we divide the return time series *R*_*i*_(*t*, *τ*) of each stock *i* into 242 time series, which are *R*_*i*_(*t*, 9: 30), *R*_*i*_(*t*, 9: 31), *R*_*i*_(*t*, 9: 32), ⋯, and *R*_*i*_(*t*, 15: 00). These time series are denoted by $R_{i}^{\tau }\left(t\right)$. For each minute *τ*, we compute the cross-correlation matrix *C*^*τ*^, of which each element is

$$C_{ij}^{\tau }=\frac{{\left\langle R_{i}^{\tau }\left(t\right)R_{j}^{\tau }\left(t\right)\right\rangle }_{t}-{\left\langle R_{i}^{\tau }\left(t\right)\right\rangle }_{t}{\left\langle R_{j}^{\tau }\left(t\right)\right\rangle }_{t}}{\sigma_{R_{i}^{\tau }}\sigma_{R_{j}^{\tau }}}.$$ (1)

Here 〈⋯〉_*t*_ represents the average over *t*, and $\sigma_{R_{i}^{\tau }}$ is the standard deviation of time series $R_{i}^{\tau }\left(t\right)$. $C_{ij}^{\tau }$ is the correlation between the returns of the *i*-th and *j*-th stocks at minute *τ*. In total, there are 242 matrix *C*^*τ*^. In this paper, the strength of the interaction of two stocks is measured by the correlation between the two stocks. Thus *C*^*τ*^ contains all the stock interactions at minute *τ*. In order to study the market state, we investigate the co-movement of stock prices, interactions of sectors and correlation between the stock interactions at different minutes.

### Stock correlation and price co-movement

**Fig 1:**
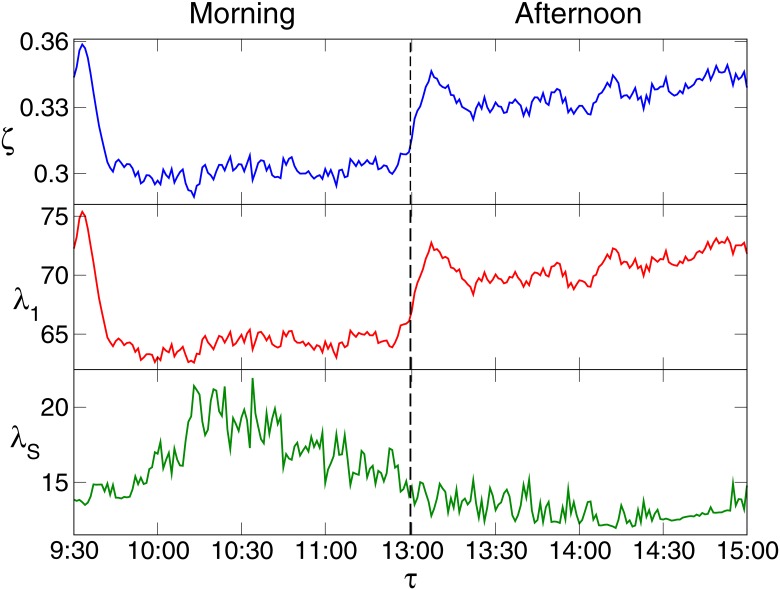
Average equal-time correlation *ζ*(*τ*) of all stocks and eigenvalues of the market and sector modes. *λ*_1_(*τ*), the largest eigenvalue, represents the market mode, and *λ*_*S*_(*τ*) stands for the sector mode. The stock market is open from 9:30 to 11:30 in the morning, and from 13:00 to 15:00 in the afternoon. The minute *τ* = 11: 30 is next to *τ* = 13: 00. For clarity, *τ* = 11: 30 is not marked in the figure.

To measure the strength of the stock interactions in the whole market, we calculate the average correlation *ζ*(*τ*) of all stocks at minute *τ*,

$$\zeta \left(\tau \right)=\frac{1}{N(N-1)}\sum_{i,j,i\neq j}C_{ij}^{\tau },$$ (2)

where *N* is the number of the stocks. As displayed in Fig 1, *ζ*(*τ*) is about 0.35 for the first 5 minutes, and sharply decreases to 0.3 at 9: 44. The large average correlation of stocks for the first 5 minutes may result from the call auction, from 9: 15 to 9: 25, before the Shanghai stock market opens. In the call auction, investors trade according to their estimation of the stock performance, and the opening price of a stock is determined by the trade with largest trading volume. After 9: 44, *ζ*(*τ*) remains basically unchanged in the morning. From the first minute in the afternoon, *ζ*(*τ*) increases sharply to about 0.34 in 6 minutes and remains stable for the rest time of the afternoon. Generally, the average stock correlation *ζ*(*τ*) is at two different levels respectively in the morning and afternoon, and *ζ*(*τ*) in the afternoon is 10 percent larger than that in the morning. This result indicates that the market states in the morning and afternoon may be different.

To further study the dynamics of the stock interactions, we calculate the eigenvalues of *C*^*τ*^, and denote the *α*-th largest eigenvalue by *λ*_*α*_(*τ*). A number of large *λ*_*α*_(*τ*) significantly deviate from the upper bound of the eigenvalue distribution of the Wishart matrix, which is the cross-correlation matrix of non-correlated time series. *λ*_1_(*τ*) represents the market mode, i.e., the price co-movement at the market level. The average stock correlation *ζ*(*τ*) mentioned above stands for the sum of all modes, including the market mode, sector modes and other modes. Since *λ*_1_(*τ*) is much larger than the other eigenvalues, the market mode is dominating. Therefore *λ*_1_(*τ*) behaves almost the same as *ζ*(*τ*). Each of the other large eigenvalues of *C*^*τ*^ represents a sector mode, i.e., the price co-movement at the sector level. Among these eigenvalues, the sector structure in the sector mode is more significant for *λ*_2_(*τ*), *λ*_3_(*τ*), *λ*_4_(*τ*) and *λ*_5_(*τ*). Thus the sector modes that these eigenvalues correspond to are the four main sector modes. Here we consider the four eigenvalues, and define the eigenvalue of the sector mode as $\lambda_{S}\left(\tau \right)=\sum_{\alpha =2}^{5}\lambda_{\alpha }\left(\tau \right)$. As shown in Fig 1, *λ*_1_(*τ*) and *λ*_*S*_(*τ*) are respectively larger in the afternoon and morning. This result indicates that the price co-movement at the market level is more significant in the afternoon than that in the morning, while the co-movement at the sector level is much stronger in the morning. *λ*_*S*_(*τ*), to be more specific, is the degree that the prices of stocks in a sector tending to rise and fall at the same time, and thus stands for the correlation of the stocks in a sector. If *λ*_*S*_(*τ*) is large, the correlation is strong. When building an investment portfolio with various stocks to avert risk, one should consider the correlation between stocks. The difference between *λ*_*S*_(*τ*) in the morning and afternoon may be helpful to investors in building investment portfolios [50].

### Sector interactions

The interactions of sectors are comprised of global interactions of sectors, local interactions of sectors and random interactions of sectors, among which the global sector interactions and local sector interactions are respectively extracted from the market and sector modes [31]. Compared to the global sector interactions, the local sector interactions are higher-order interactions, through which we can observe the fine structure of sectors.

Due to the fluctuation of the local sector interactions of the cross-correlation matrix *C*^*τ*^ in one minute, we average *C*^*τ*^ over *τ* within a time window *T*. If *T* is too small, e.g. 10 minutes, the fluctuation is still large. In one trading day, there are respectively two hours in the morning and afternoon. In order to investigate the evolution of the local sector interactions in the morning, in the afternoon and between the morning and afternoon, we set *T* = 1 hour. The four hours in one trading day are respectively from 9: 30 to 10: 30, from 10: 31 to 11: 30, from 13: 00 to 13: 59 and from 14: 00 to 15: 00. For the *γ*-th hour, each element of the average cross-correlation matrix $\tilde{C}\left(\gamma \right)$ is $\tilde{C_{ij}}\left(\gamma \right)={\langle C_{ij}^{\tau }\rangle }_{\tau }{|}_{\tau \in \gamma -\text{th hour}}$. Here 〈⋯〉_*τ*_ represents the average over *τ*. We adopt the method in Ref. [31], which combines the RMT, PMFG and Infomap, to capture the local sector interactions from $\tilde{C}\left(\gamma \right)$ for each hour.

We denote the *α*-th largest eigenvalue of matrix $\tilde{C}\left(\gamma \right)$ by ${\tilde{\lambda }}_{\alpha }\left(\gamma \right)$, and the *i*-th component of the corresponding eigenvector by ${\tilde{u}}_{i}^{\alpha }\left(\gamma \right)$. According to the RMT, a matrix $\tilde{C}\left(\gamma \right)$ can be decomposed into *N* different modes,

$$\tilde{C_{ij}}\left(\gamma \right)=\sum_{\alpha =1}^{N}{\tilde{\lambda }}_{\alpha }\left(\gamma \right){\tilde{u}}_{i}^{\alpha }\left(\gamma \right){\tilde{u}}_{j}^{\alpha }\left(\gamma \right).$$ (3)

We consider the eigenvalues for the four main sector modes, i.e., ${\tilde{\lambda }}_{2}\left(\gamma \right)$, ${\tilde{\lambda }}_{3}\left(\gamma \right)$, ${\tilde{\lambda }}_{4}\left(\gamma \right)$ and ${\tilde{\lambda }}_{5}\left(\gamma \right)$. Each element of the matrix ${\tilde{C}}^{sec}\left(\gamma \right)$ of the sector mode is defined as

$${\tilde{C_{ij}}}^{sec}\left(\gamma \right)=\sum_{\alpha =2}^{5}{\tilde{\lambda }}_{\alpha }\left(\gamma \right){\tilde{u}}_{i}^{\alpha }\left(\gamma \right){\tilde{u}}_{j}^{\alpha }\left(\gamma \right).$$ (4)

Next, a network is constructed from $|{\tilde{C}}^{sec}\left(\gamma \right)|$ with the PMFG method. The Infomap method is applied to obtain the main interaction structure of communities from the network, and a map of the community structure is generated.

**Fig 2:**
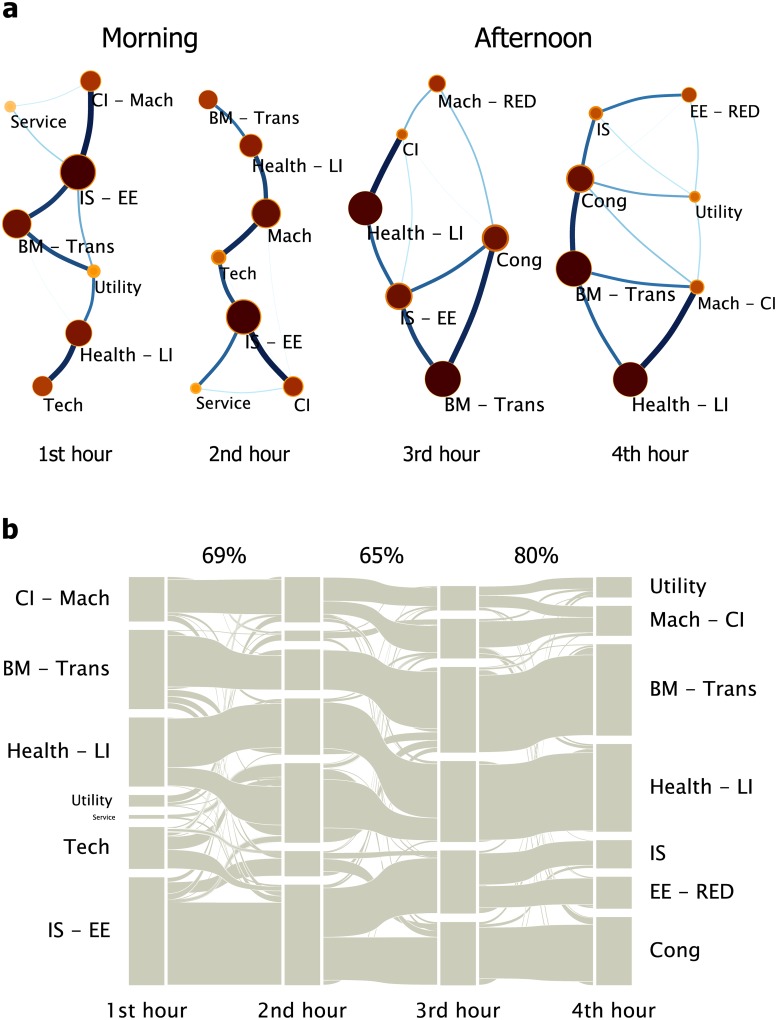
(a) The structure of local sector interactions extracted from the sector mode and (b) the evolution of the component stocks of sectors for the four hours. The first and second two hours are respectively in the morning and afternoon. The importance of each stock is different. For subfigure (a), a circle is a community comprised of stocks, and the line connecting two communities represents the interactions between them. A community is named after the sector or sectors of its most important stocks. For subfigure (b), the communities are arranged in column for each hour. The line connecting two communities in two adjacent hours represents the flow of stocks. A flow is thicker if the stocks in the flow are more important. The overlapping percentages of sectors during the evolution are displayed above the figure.

According to the Infomap method, the importance of each stock is different. The more information flows past a stock, the more important the stock is. As shown in Fig 2(a), a circle in a map is a community comprised of stocks. The more important a community is in the network, the bigger the circle is. The line connecting two circles is thicker if the interaction of the two communities is stronger. In the dynamics of complex financial systems, a community is also called an industry sector, since the stocks in a community usually share common economic properties. The sector named “IS—EE”, for example, is mainly comprised of subsectors “Information service (IS)” and “Electronic elements (EE)”. Thus, the maps of community structure in Fig 2(a) are exactly the maps of the sector structure.

In Fig 2(a), we observe that the interaction structure of sectors evolves with time. The two sector structures in the afternoon are both net-like structure, while those in the morning are more closed to linear structure. Thus, the sector structure in the afternoon is more complicated. The net-like or linear appearances of the structures in the morning and afternoon are robust if *C*^*τ*^ is averaged over *T* = 2 hours, and basically the same for *T* = 0.5 hour, with some fluctuations. According to Ref. [31], the sector structure of a stock market in the financial crisis period is less complicated than that in the normal period. It indicates that when a market is vulnerable, its structure becomes simpler. In other words, in complex financial systems, a complicated structure is firmer. Therefore the sector structure in the afternoon is firmer than that in the morning.

For a sector, the component stocks, i.e. the stocks that comprise it, are different at each hour. The evolution of the component stocks of sectors for the four hours is displayed in Fig 2(b). During the evolution, the two sectors, “BM—Trans” and “Health—LI”, seem to be the most robust sectors, of which the component stocks basically remain. To quantify the similarity between the sectors in two adjacent maps, we propose a technique to calculate the overlapping percentage of the component stocks of the sectors in the two maps (see S1 Text). 69 percent of the component stocks of sectors overlap for the two hours in the morning, while the value is 80 percent in the afternoon. Between the morning and afternoon, the overlapping percentage is 65 percent. Therefore, the sectors in the two hours in the afternoon are more similar in component stocks, i.e., the component stocks of sectors are more robust in the afternoon. This result basically remains robust for *T* = 0.5 hour.

In summary, the component stocks of sectors are more robust and the structure of sectors is firmer in the afternoon. Therefore, the market state in the afternoon is more stable than that in the morning.

### Correlation between stock interactions at different minutes

The cross-correlation matrix *C*^*τ*^ contains all the stock interactions at minute *τ*. In the previous two subsections, we have investigated the stock interactions and sector interactions, including the average stock correlation, price co-movement, sector structure and component stocks of sectors. However, our knowledge is very limited on the correlation among the stock interactions at different minutes, where certain patterns may exist. To search for these patterns, we construct the cross-correlation matrix *M* of *C*^*τ*^, and further investigate the co-movement modes of *C*^*τ*^ at different minutes in one trading day. Each element of *M* is

$$M_{\tau \tilde{\tau }}={\left.\frac{{\left\langle C_{ij}^{\tau }C_{ij}^{\tilde{\tau }}\right\rangle }_{i,j}-{\left\langle C_{ij}^{\tau }\right\rangle }_{i,j}{\left\langle C_{ij}^{\tilde{\tau }}\right\rangle }_{i,j}}{\sigma_{C_{ij}^{\tau }}\sigma_{C_{ij}^{\tilde{\tau }}}}\right|}_{i\neq j}.$$ (5)

Here 〈⋯〉_*i*, *j*_ represents the average over *i* and *j*, and $\sigma_{C_{ij}^{\tau }}$ is the standard deviation of all the elements in $C_{ij}^{\tau }$ except those on the diagonal. $M_{\tau \tilde{\tau }}$ represents the correlation between the stock interactions at minute *τ* and $\tilde{\tau }$. Thus, *M* is not the same as other correlation matrices in previous research studies [11, 12, 21–26], which are constructed of equal-time correlations. *M* contains all the correlation between the stock interactions at two different minutes.

**Fig 3:**
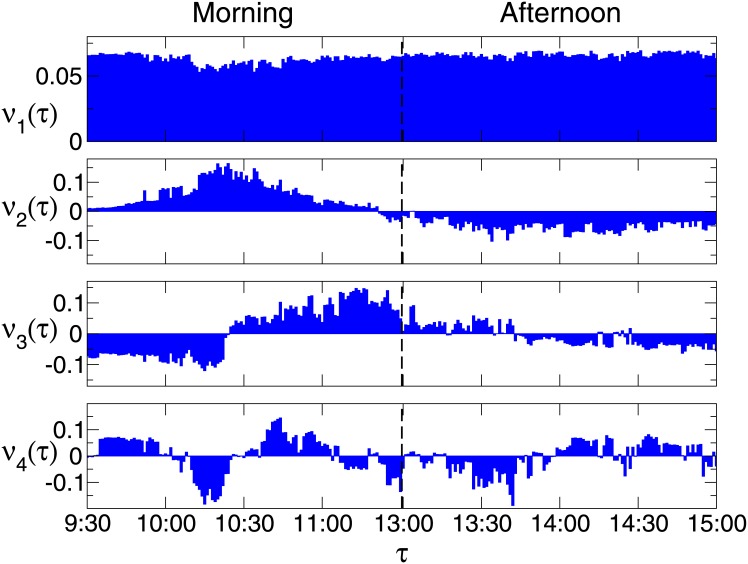
The eigenvectors of the four largest eigenvalues of the cross-correlation matrix *M*.

The *α*-th largest eigenvalue of *M* is denoted by $\lambda_{\alpha }^{M}$. For simplicity, we call the first largest eigenvalue the first eigenvalue, and so on. The first three eigenvalues of *M* are respectively 199.26, 5.72 and 4.11. Since the first eigenvalue $\lambda_{1}^{M}$ is much larger than the other *M* − 1 eigenvalues, the co-movement mode described by the first eigenvector is dominating. For $\lambda_{\alpha }^{M}$, the *τ*-th component of the eigenvector is denoted by *ν*_*α*_(*τ*). As displayed in Fig 3, the components *ν*_1_(*τ*) are almost uniform for all *τ*, indicating that the first co-movement mode of *C*^*τ*^ is basically the same for different *τ*. The uniformity in *ν*_1_(*τ*) is quite similar to that in the first eigenvector of the cross-correlation matrix in previous research studies [11, 12, 21–26]. We may call $\lambda_{1}^{M}$ the market mode of matrix *M*, and it represents the collective movement of *C*^*τ*^ at all minute *τ*.

In previous works, for the eigenvector corresponding to a sector mode, the components for stocks in a sector are usually of a same sign and similar values. Here we define a sector in an eigenvector of matrix *M* as the fragment of *ν*_*α*_(*τ*) with a same sign and successive *τ*. For the eigenvector of the second eigenvalue of *M*, the components *ν*_2_(*τ*) in the morning are mainly positive, while those in the afternoon are negative. Thus, we call them a “morning” sector and an “afternoon” sector, respectively. Note that for an eigenvector, the physical meaning of the sign of a component is in a relative sense, i.e., the sign of a component is meaningless, unless it is compared with the sign of another component. The second co-movement mode of *C*^*τ*^, characterized by the second eigenvector, comprises the “morning” sector and “afternoon” sector. These two sectors are anti-correlated, and we call them a “sector pair”. Thus we may call the second co-movement mode the “day” sector pair. Compared with the first co-movement mode, the second one is a fine co-movement mode, and the anti-correlation is within this mode. Taking into account the absolute values of the components *ν*_2_(*τ*), we observe that the values in the morning are larger, suggesting that the co-movement in the morning is stronger.

For the eigenvector of the third eigenvalue, the co-movement mode is dominated by the movement in the morning, since the absolute values of the components in the morning are much larger than those in the afternoon. This mode is mainly comprised of an anti-correlated sector pair, which is in the morning, and we call the mode the “morning” sector pair. From the eigenvectors of the forth eigenvalue, we can hardly identify a co-movement pattern of *C*^*τ*^.

To test the robustness of the results in Figs 1, 2 and 3, we divide the time series of all stocks into two parts, a bull market part and a bear market part, according to the financial crisis on July 20, 2001 (see S2 Text). The results in Figs 1, 2(a) and 3 are robust for the both parts. For the results in Fig 2(b), in the bear market part, the component stocks of sectors in the afternoon are more robust than those in the morning. In the bull market part, the component stocks of sectors in the morning are as robust as those in the afternoon (see S2 Text). Considering the result of the sector structure and that of the component stocks, we find that for both parts, the market state is more stable in the afternoon.

We have shown in the first three subsections that the market states in the morning and afternoon are significantly different. The differences mainly exist in the co-movement of stock prices, interactions of sectors and correlation between the stock interactions at different minutes. In the afternoon, the component stocks of sectors are more robust and the structure of sectors is firmer. Therefore, the market state in the afternoon is more stable.

### Indicator for financial crises

Understanding financial crises is important for the risk estimation of investment, and many previous activities have been devoted to the financial crises [40–46]. In this subsection, we will illustrate that the information of the sector interactions can indicate the financial crisis in the Shanghai stock market, and the indicator based on the empirical data in the afternoon is more effective.

**Fig 4:**
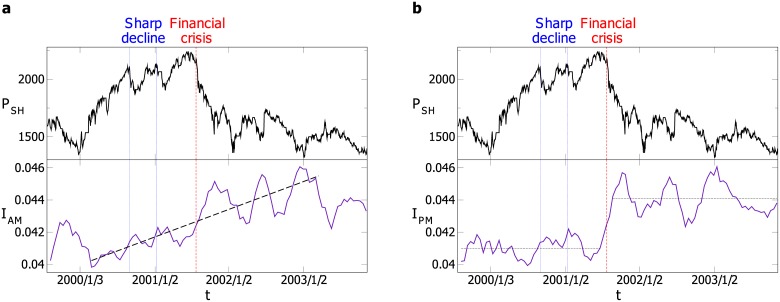
The Shanghai Index and indicator (a) *I*_*AM*_ (b) *I*_*PM*_. For both (a) and (b), the two vertical dash lines on the left respectively denote the beginnings of two sharp declines of the Shanghai Index, and the one on the right denotes the start of the financial crisis. The dash lines for *I*_*AM*_ and *I*_*PM*_ are the guild lines for the trend of the indicator in corresponding periods.

The daily closing price of the Shanghai Index on day *t* is denoted by *P*_*SH*_(*t*). A financial crisis is the situation in which a financial market suddenly lose a large part of its value. In this paper, we simply define the financial crisis in the Shanghai stock market as a period shorter than 6 months, during which *P*_*SH*_(*t*) declines more than 30 percent. We select a large value of *P*_*SH*_(*t*) before the decreasing trend as the beginning of the financial crisis. The period and beginning are determined qualitatively by visual observation. However, a little alteration in the period or beginning does not affect the robustness of our results in this subsection. As displayed in Fig 4, from July 20, 2001 to January 18, 2002, *P*_*SH*_(*t*) decreases 35.1 percent. We consider this period as a financial crisis. Before the financial crisis, there are two sharp declines, during which *P*_*SH*_(*t*) declines 10 percent.

We denote a period of time in one trading day, specifically the morning and afternoon, by *X*. Periods *X* = a.m. and *X* = p.m. respectively represent the morning and afternoon. There are 1013 days in the return time series of each stock. Omitting the returns in the last 3 days, we divide the time series into 101 parts with a 10-day moving window without overlapping. For all the time periods *X* in the *k*-th part, each element of the equal-time cross-correlation matrix *C*^*X*^(*k*) of all stocks is

$$C_{ij}^{X}\left(k\right)={\left.\frac{{\left\langle R_{i}\left(t,\tau \right)R_{j}\left(t,\tau \right)\right\rangle }_{t,\tau }-{\left\langle R_{i}\left(t,\tau \right)\right\rangle }_{t,\tau }{\left\langle R_{j}\left(t,\tau \right)\right\rangle }_{t,\tau }}{\sigma_{R_{i}}\sigma_{R_{j}}}\right|}_{t\in k-\text{th part},\;\tau \in X}.$$ (6)

Here 〈⋯〉_*t*, *τ*_ represents the average over *t* and *τ*, and *σ*_*R*_*i*__ is the standard deviations of *R*_*i*_(*t*, *τ*). For matrix *C*^*X*^(*k*), we denote the *α*-th largest eigenvalue by $\lambda_{\alpha }^{X}$, and the *i*-th component of the corresponding eigenvector by $u_{\alpha }^{X}\left(i\right)$. We only consider the eigenvalues and eigenvectors of the four main sector modes, i.e. *α* = 2, 3, 4 and 5, which contain information of sector interactions. The standard deviation of eigenvector $|u_{\alpha }^{X}|$ is denoted by $\sigma_{u}^{X}\left(\alpha \right)$. For an eigenvector $u_{\alpha }^{X}$, if the absolute values of the components in a same sector are significantly larger than those of other components, the sector structure described by this eigenvector is significant. In this case, $\sigma_{u}^{X}\left(\alpha \right)$ would be large. Otherwise, $\sigma_{u}^{X}\left(\alpha \right)$ would be small. Thus for eigenvector $u_{\alpha }^{X}$, the standard deviation $\sigma_{u}^{X}\left(\alpha \right)$ is a simple indicator for the significance of the sector structure. We define the indicators *I*_*X*_ for time period *X* as

$$I_{X}\left(k\right)=\sum_{\alpha =2}^{5}\left[\lambda_{\alpha }^{X}/\lambda_{S}^{X}\cdot \sigma_{u}^{X}\left(\alpha \right)\right].$$ (7)

Here $\lambda_{S}^{X}=\sum_{\alpha =2}^{5}\lambda_{\alpha }^{X}$. The indicators *I*_*X*_ with *X* = a.m. and *X* = p.m. are respectively denoted by *I*_*AM*_ and *I*_*PM*_. Then, *I*_*AM*_ and *I*_*PM*_ are smoothed with a 5-point moving window. The daily closing price of the Shanghai Index on day *t* is denoted by *P*_*SH*_(*t*).

The indicators are displayed with *P*_*SH*_(*t*) in Fig 4. When the financial crisis occurs, both *I*_*AM*_ and *I*_*PM*_ increase significantly, and are generally much larger in the financial crisis period. The Shanghai Index sharply declines twice before the financial crisis, and the indicators remain basically unchanged or decrease. It suggests that these two indicators are capable of indicating the financial crisis. As shown in Fig 4(b), when or even before the financial crisis happens, *I*_*PM*_ increases sharply to a new level, which is significantly higher than the level before the crisis. During the financial crisis, *I*_*AM*_ rises gradually. Since *I*_*PM*_ is less fluctuating and changes suddenly when the financial crisis occurs, *I*_*PM*_ is more able to discriminate the financial crisis from other sharp declines. Therefore, *I*_*PM*_ is a better indicator than *I*_*AM*_. In other words, the indicator based on the empirical data in the afternoon is more effective in indicating the financial crisis. This may result from that the market is in a more stable state in the afternoon. The indicator *I*_*AM*_ is based on the information of the sector interactions in the morning. Since the component stocks of sectors are less robust and the structure of sectors is less firm in the morning, *I*_*AM*_ may contain much randomness, leading it to be less effective.

According to the previous research study [15], the equal-time cross-correlation between stocks is stronger during a financial crisis. Therefore, the interactions within and between sectors in a financial crisis differ from those in a normal period. The indicator *I*_*PM*_ captures the sector interactions, and thus can indicate the financial crisis.

## Discussion

A stock market is a non-stationary complex system, and the market state evolves with time. The stock interactions is important for the understanding of the market state. However, our knowledge on the stock interactions on the minute timescale is limited.

Based on 50 million minute-to-minute data in the Shanghai stock market, we discover that the market states in the morning and afternoon are significantly different. The differences mainly exist in three aspects, i.e. the co-movement of stock prices, interactions of sectors and correlation between the stock interactions at different minutes. We observe that the average equal-time correlation *ζ*(*τ*) of all stocks is at two different levels respectively for the morning and afternoon. The price co-movement at the sector level is more significant in the morning, while that at the market level is much stronger in the afternoon. By analyzing the interactions of sectors, we detect that in the afternoon, the component stocks of sectors are more robust and the structure of sectors is firmer. Therefore, the market state in the afternoon is more stable. We construct the cross-correlation matrix of *C*^*τ*^ to investigate the correlation between the stock interactions at different minutes within one trading day, specifically the co-movement modes of the stock interactions. The first co-movement mode of *C*^*τ*^ is basically the same for all minutes. The second mode of *C*^*τ*^, which is a fine co-movement mode, comprises a “morning” sector and “afternoon” sector. These two sectors are anti-correlated, and form a “sector pair”.

Furthermore, our results reveal that the information of the sector interactions can indicate the financial crisis in the market, and the indicator based on the empirical data in the afternoon is more effective. This may result from that the market is in a more stable state in the afternoon.

## Supporting Information

S1 Text Overlapping percentage of the component stocks of sectors. A technique is proposed to quantify the overlapping percentage between two adjacent maps.
(PDF)

S2 Text Sector interactions in bull market and bear market periods. The time series of all stocks are divided into a bull market part and a bear market part, and the sector interactions are investigated for the two parts.
(PDF)
