# Supplementary material for: Exploring Market State and Stock Interactions on the Minute Timescale
Source: PLoS One. 2016 Feb 22;11(2):e0149648. doi: 10.1371/journal.pone.0149648 (PMC4762888; doi:10.1371/journal.pone.0149648)
Supplement: S1 Text — A technique is proposed to quantify the overlapping percentage between two adjacent maps. (PDF) [file pone.0149648.s001.pdf]

S1 Text

Overlapping percentage of the component stocks of sectors

We propose a technique to quantify the overlapping percentage of the component stocks of sectors between two adjacent maps. To illustrate the technique, we take Fig. 1 as an example. In a structure map of sectors, the importance of the k-th stock is denoted by S_k. S_k is different for each stock. A sector is comprised of stocks, and the importance of the i-th sector is denoted by M_i,

$$M_i = \sum_k S_k \Big|_{k\text{-th stock}\in i\text{-th sector}}.$$

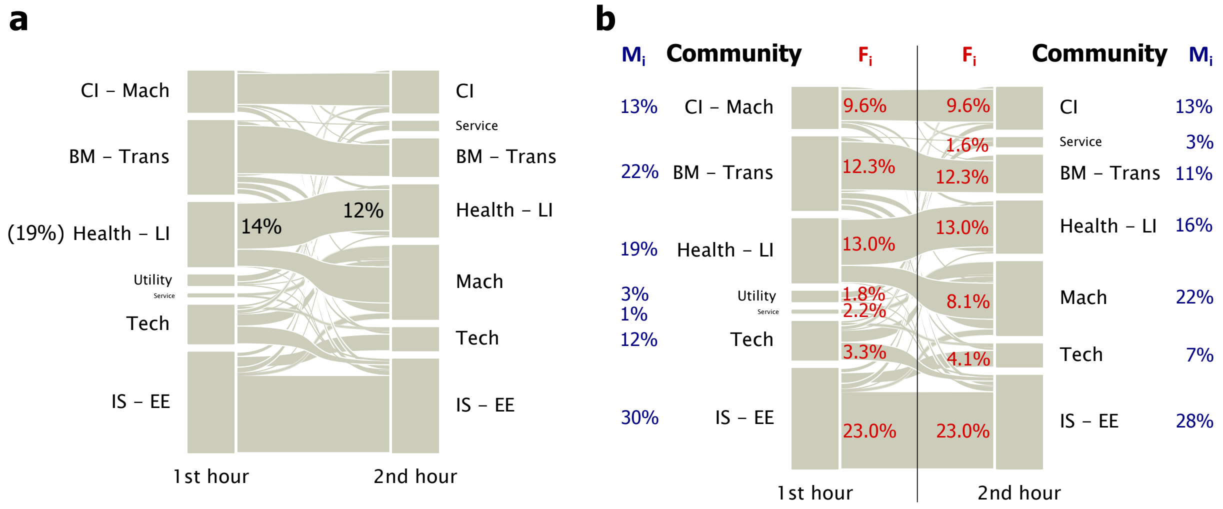

Figure 1: **Evolution of the component stocks of sectors for the 1st and 2nd hours.**

As displayed in Fig. 1(a), there are 7 sectors in the 1st hour map. From the top down, the third sector is "Health - LI", and its importance M_3 is 19 percent. The importance of the main flow of this sector is 14 percent. Since the importance of a stock is not the same for different maps, the importance of the main flow is 12 percent in the 2nd hour map. We define the importance of the main flow of the i-th sector as

$$F_i = \left(\sum_k S_k \Big|_{\text{1st hour map; } k\text{-th stock}\in\text{flow}} + \sum_k S_k \Big|_{\text{2nd hour map; } k\text{-th stock}\in\text{flow}} \right) /2,$$

and the overlapping percentage of this sector as

$$P_i = F_i / M_i.$$

For example, the importance F_3 of the main flow of "Health - LI" in the 1st hour map is $F_3 = (14\% + 12\%)/2 = 13\%$, and the overlapping percentage is $P_3 = F_3/M_3 = 13\%/19\%$.

All the importance of sectors and main flows is displayed in Fig. 1(b). We first only consider the sectors in the 1st hour map. Taking the importance M_i of sectors as the wight, we calculate the overlapping percentage from the left side

$$P_{left} = \sum_{i=1}^{7} M_i P_i \Big|_{\text{1st hour map}} = \sum_{i=1}^{7} F_i \Big|_{\text{1st hour map}}.$$

Then we consider the right side, i.e. the 2nd hour map,

$$P_{right} = \sum_{i=1}^{7} F_i \bigg|_{\text{2nd hour map}} .$$

Thus the overlapping percentage of the component stocks of sectors between the two maps is

$$P = (P_{left} + P_{right})/2.$$
