# Supplementary material for: Exploring Market State and Stock Interactions on the Minute Timescale
Source: PLoS One. 2016 Feb 22;11(2):e0149648. doi: 10.1371/journal.pone.0149648 (PMC4762888; doi:10.1371/journal.pone.0149648)
Supplement: S2 Text — The time series of all stocks are divided into a bull market part and a bear market part, and the sector interactions are investigated for the two parts. (PDF) [file pone.0149648.s002.pdf]

S2 Text

Sector interactions in bull market and bear market periods

Each stock of our data is from July 27, 1999 to November 5, 2003. During this period, a financial crisis occurs in the Shanghai stock market on July 20, 2001. The daily closing price of the Shanghai Index on day t is denoted by $P_{SH}(t)$. As shown in Figure 1, $P_{SH}(t)$ sharply declines from 2200 to 1400 when the financial crisis happens. To test the robustness of the results in the manuscript, we divide the time series of all stocks into two parts, a bull market part and a bear market part, according to the financial crisis.

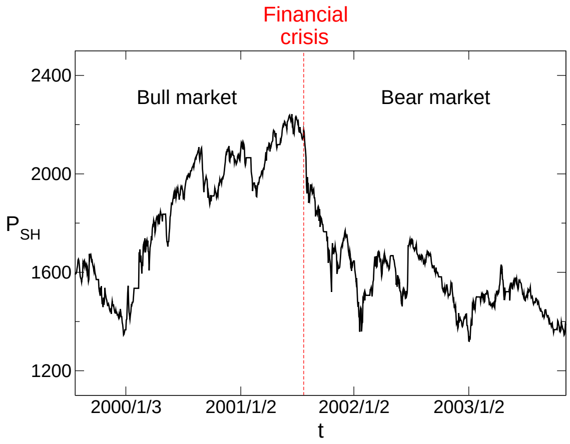

Figure 1: **The bull market and bear market periods of the Shanghai stock market.**

The results of the sector interactions for the two parts are respectively displayed in Figure 2 and 3. In the bear market part, as shown in Figure 3(b), the component stocks of sectors in the afternoon are more robust than those in the morning. This result is the same as that of the whole time series in the manuscript. In the bull market part, the component stocks of sectors in the morning are as robust as those in the afternoon. For both the bull market and bear market parts, the sector structure in the afternoon is more complicated than those in the morning. Therefore, the market state is more stable in the afternoon for the two parts.

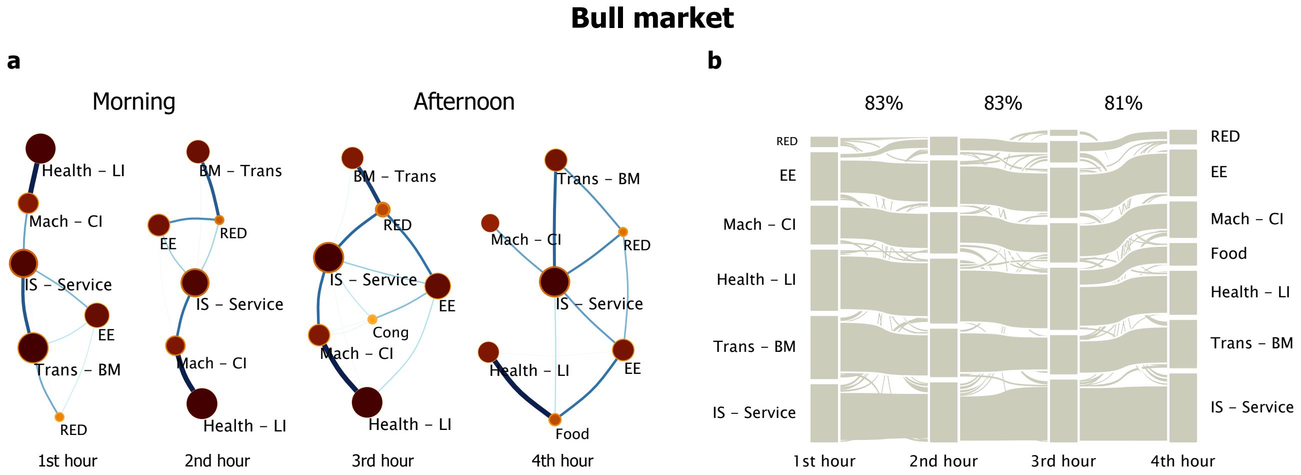

Figure 2: **(a) Sector structure in the sector mode and (b) evolution of the component stocks of sectors for the bull market part.** The first and second two hours are respectively in the morning and afternoon. For subfigure (b), the overlapping percentage of sectors during the evolution is displayed above the figure.

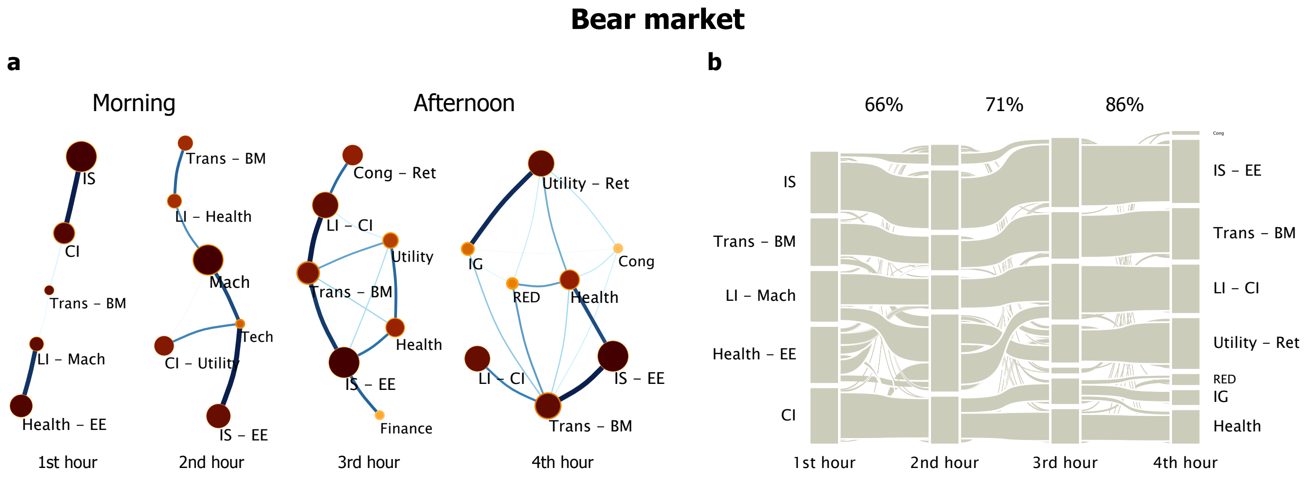

Figure 3: **(a) Sector structure in the sector mode and (b) evolution of the component stocks of sectors for the bear market part.**
